# An Uncommon Cause of Posterior Reversible Encephalopathy Syndrome Related to Antibiotic Ingestion

**DOI:** 10.7759/cureus.3540

**Published:** 2018-11-03

**Authors:** Souda El-Sheikh, Muhamad Memon, Ahmed Mujtaba, Humariya Heena

**Affiliations:** 1 Neurology, King Fahad Medical City, Riyadh, SAU; 2 Internal Medicine, King Fahad Medical City, Riyadh, SAU; 3 Epidemiology and Public Health, King Fahad Medical City, Riyadh, SAU

**Keywords:** posterior reversible encephalopathy syndrome, eruptive skin lesions, amoxicillin and clavulanic acid

## Abstract

Posterior reversible encephalopathy syndrome (PRES) is a neurotoxic state manifested with a unique computed tomography (CT) or magnetic resonance imaging (MRI) appearance. PRES is associated with different conditions, such as eclampsia, sepsis, organ transplantation, and drugs, especially immunosuppressive medications. Besides pharmacologic side effects, antibiotics can cause PRES as well.

Here, we report a 37-year-old female from Saudi Arabia, presenting to the emergency department (ED) with a two-day history of fluctuations in consciousness level, headache, and blurring of vision. A generalized vesicular skin rash preceded the condition for one month; this was diagnosed as chicken pox and the patient received co-amoxiclav for a possible superadded bacterial infection.

Besides clinical manifestations, the patient had radiological abnormalities, which were resolved following the withdrawal of causative antibiotics. Ascertaining the exact etiological cause of PRES is essential for diagnosing this reversible condition as these patients undergo a complete neurological recovery if the underlying cause is identified early.

## Introduction

Posterior reversible encephalopathy syndrome (PRES) is a neurotoxic state manifested with a unique computed tomographic (CT) or magnetic resonance imaging (MRI) appearance. Many pathological conditions and treatments have been associated with this syndrome. Ascertaining the exact etiological cause of PRES is essential for diagnosing this reversible condition, as these patients undergo a complete neurological recovery if the underlying cause is identified early.

Being introduced in the mid-nineties, PRES has a classic repertoire of symptoms such as alteration of vision, altered mental state, new onset headache, seizures, and findings on brain imaging studies with typical MRI and CT changes [1]. Opinions differ regarding the most characteristic presenting feature of PRES with some studies documenting encephalopathy while others considering seizures to be the most frequently occurring (74%) symptom. However, any one of the leading features (encephalopathy, seizure, headache, and visual disturbance) can be the presenting symptom [2-3]. The pathophysiologic events leading to the development of PRES are debatable at present. PRES can occur due to hypertension, leading to hyperperfusion or due to vasoconstriction causing hypoperfusion and endothelial dysfunction/injury causing brain edema [4-5]. However, the pathophysiologic mechanism causing PRES improves with strict hemodynamic control and the elimination of the offending agent.

We report a 37-year-old woman, followed up initially in another hospital with a one-month history of eruptive skin lesions, diagnosed with chicken pox and secondary bacterial infection. She was diagnosed as a case of posterior reversible encephalopathy syndrome induced by the administration of antibiotics: amoxicillin and clavulanic acid (Augmentin). Clinical manifestations resolved entirely with the withdrawal of the causative drugs and the patient had a complete neurological recovery.

## Case presentation

A 37-year-old female from Saudi Arabia, non-smoker and non-alcoholic, with no significant history of any previous medical problems presented to the emergency department (ED) at King Fahd Medical City, Riyadh, Saudi Arabia, with a history of fluctuation in her consciousness level for two days. She had a generalized vesicular skin rash for the past one month before this presentation, which required admission to another hospital for two weeks. There, she was diagnosed with chicken pox and received local creams and oral antibiotics (Augmentin). A few days following discharge, she started complaining of a headache, blurring of vision, generalized weakness, a deteriorating level of consciousness, and a worsening skin rash.

**Figure 1:**
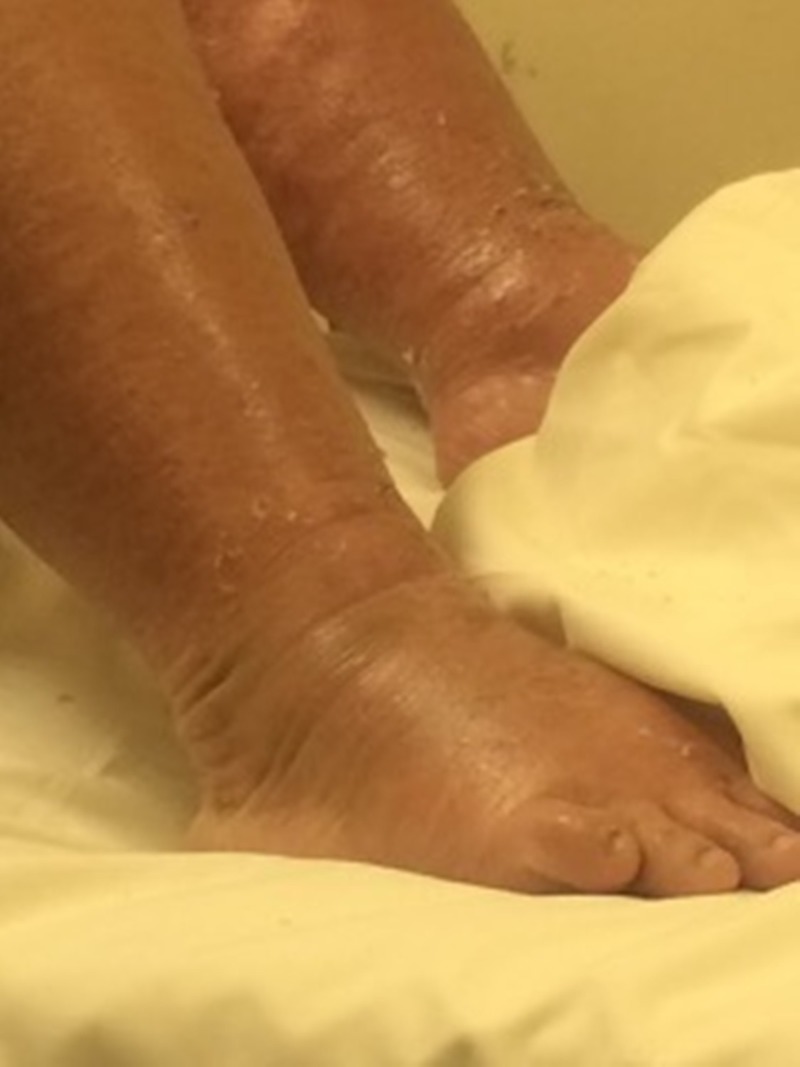
Areas of desquamation and skin peeling

Upon presentation to our ED, her Glasgow coma scale (GCS) was less than 8; she was intubated and mechanically ventilated in the emergency room. She was hemodynamically stable with a generalized maculopapular, vesicular rash all over her body with some desquamation areas (Figure 1).

The patient was admitted to the intensive care unit (ICU), intubated, ventilated, and an external ventricular drain (EVD) was inserted because of high intracranial pressure on imaging with the clinical diagnosis of bilateral posterior cerebral artery (PCA) strokes and cortical blindness.

Multiple skin biopsies were negative for vasculitis as was the hypercoagulability screen. A skin biopsy showed the presence of subepidermal vesicles, with a scanty intravesicular inflammatory cells infiltrate and linear C3 staining at the basement membrane zone, suggestive of a drug eruption with no evidence of vasculitis. The patient did not have any history of seizures, abnormal movement, behavioral changes, or any flu-like symptoms. She had multiple abortions, the last one being five months before the present illness. There was no history of a similar condition, strokes, or thrombotic events in the family or contact with a sick patient.

**Figure 2:**
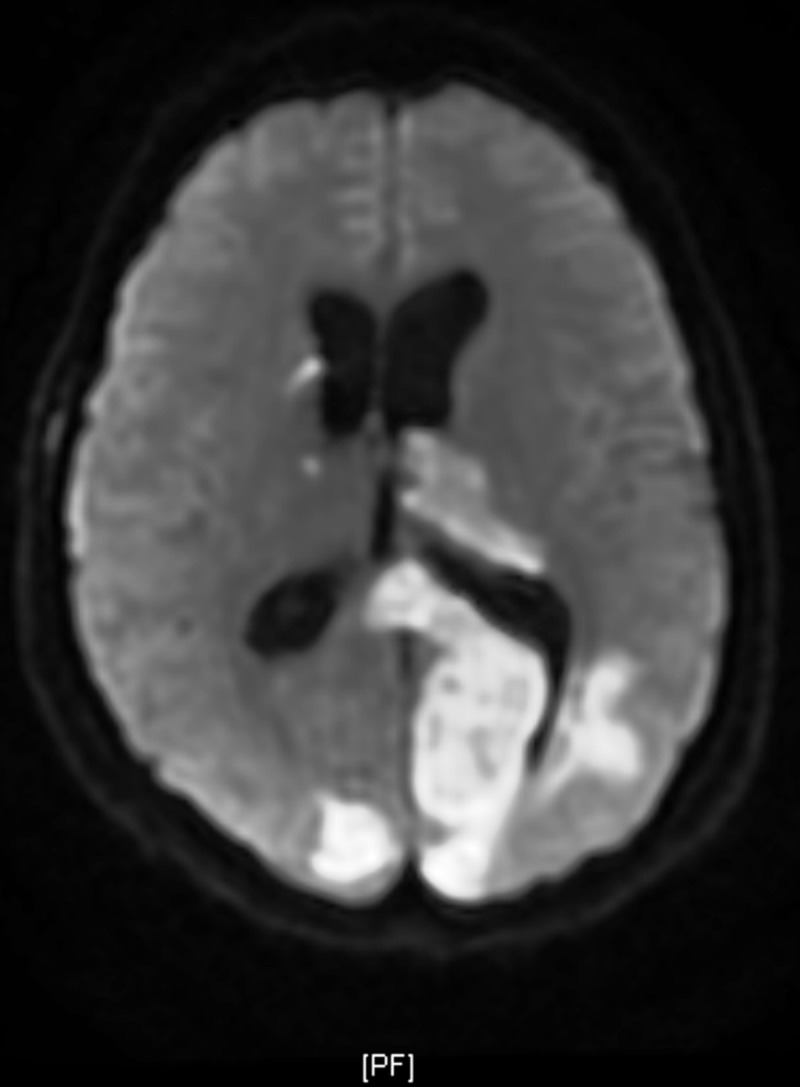
Magnetic resonance imaging (MRI) images diffusion-weighted imaging/apparent diffusion coefficient (DWI/ADC) showing an acute infarct in the left occipitoparietal, left thalamic, and right occipital region involving both the posterior cerebral artery (PCA) territories.

**Figure 3:**
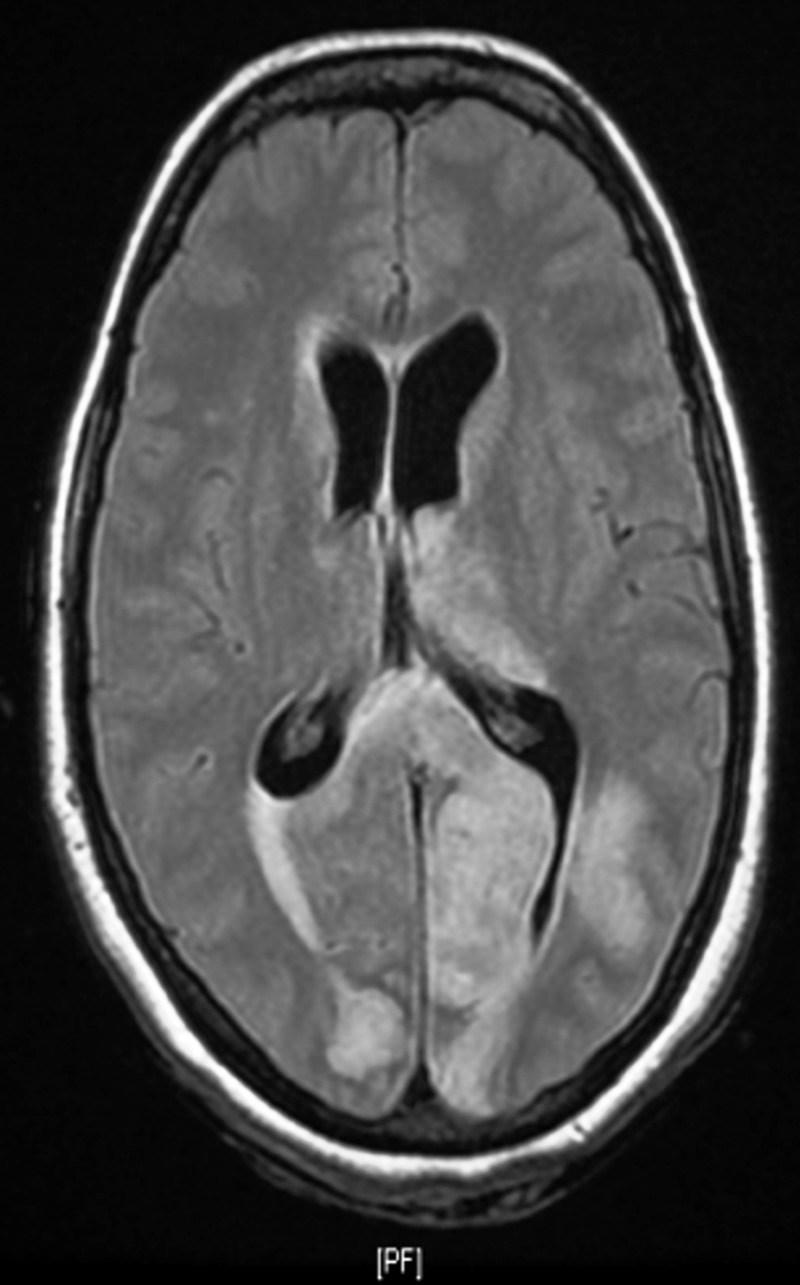
Fluid-attenuated inversion recovery (FLAIR) image of brain magnetic resonance imaging (MRI) showing hyperdensities in the cortex and subcortical white matter of the left parietooccipital lobe, right occipital consistent with posterior reversible leukoencephalopathy syndrome

Upon admission, MRI brain showed diffuse abnormal T2/fluid-attenuated inversion recovery (FLAIR), hyperintensity involving both cerebellar hemispheres, the inferior vermis, the posterior aspect of the left temporal lobe, both occipital lobes, the left thalamus, and the splenium of the corpus callosum with restricted diffusion (Figures 2-3).

A similar abnormal signal intensity involved the dorsal aspect of the pons, right middle cerebellar peduncle, and posterior aspect of the bilateral parietal lobes. These findings were consistent with acute infarction. Blooming foci in the right cerebellum hemisphere and inferior vermis denoted a micro-bleed and foci of a high T1 signal intensity within the bilateral occipital and posterior left temporal lobe, denoting microhemorrhages. There was a mass effect evident by cerebellar tonsils herniation through the foramen magnum about 28 mm, with an effacement of the basal cisterns and fourth ventricle, which resulted in supratentorial hydrocephalus and trans-ependymal permeation.

A magnetic resonance angiogram (MRA) of the brain showed a marked attenuation of the V4 segment of vertebral arteries as well as the anterior inferior cerebellar artery (AICA), superior cerebellar, and posterior cerebellar artery bilaterally with an attenuation of the supraclinoid segment of the middle cerebral artery (MCA) bilaterally. She was extubated after a few days and shifted to the high dependency unit as her conscious level improved. The patient was evaluated by the ophthalmology team and labeled to have cortical blindness related to a bilateral PCA stroke. The patient was on prednisolone oral dose of 60 mg on admission, which was gradually tapered. A meningeal biopsy was offered to the patient but refused by the family. The patient started to walk with minimal assistance; however, bilateral cortical blindness limited her mobility. She was later discharged on a tapering dose of prednisolone and aspirin 81 mg daily.

On discharge, the patient showed significant improvement in the eruptive skin rash, which dried out with some areas of crusting.

**Figure 4:**
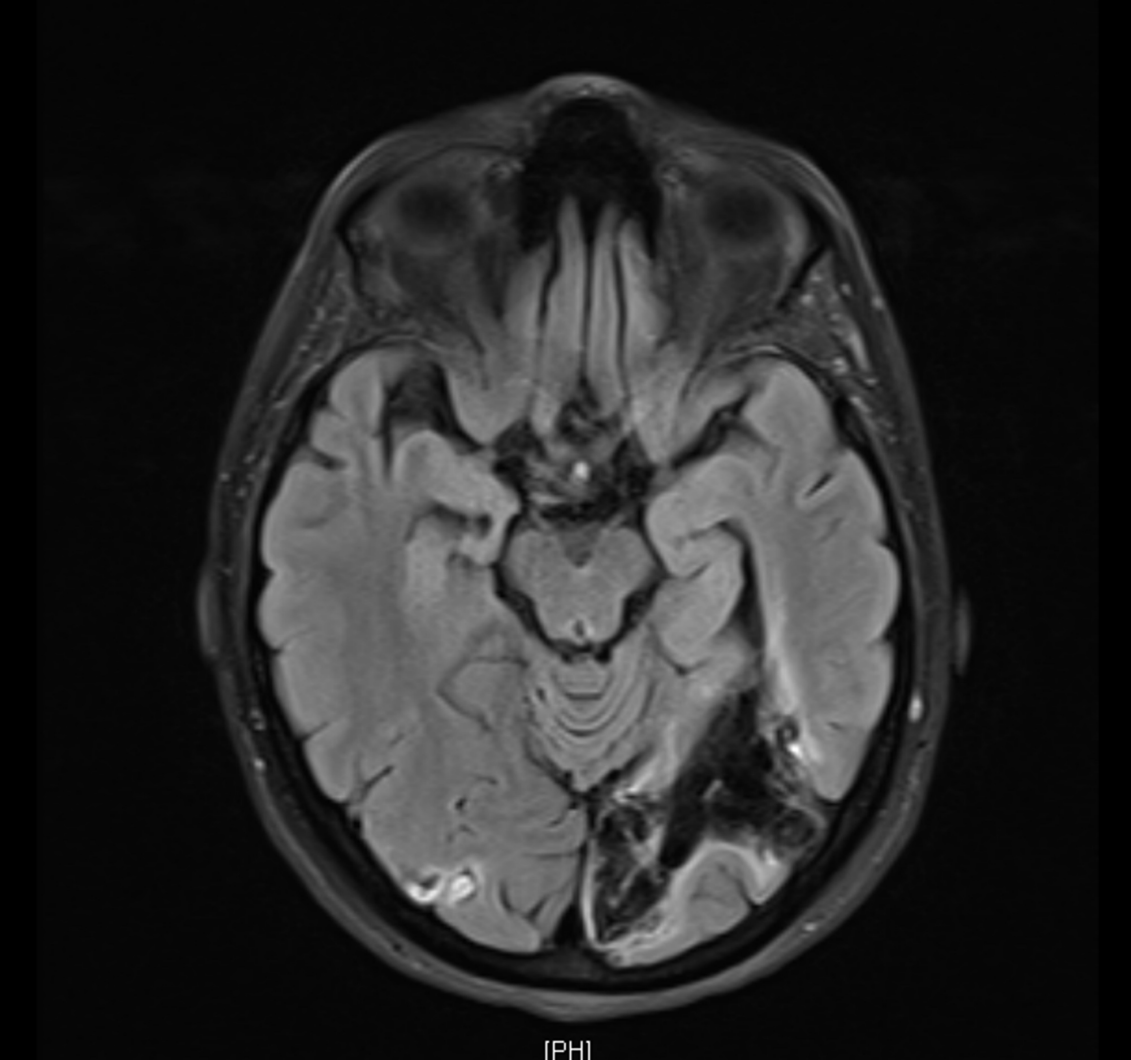
Follow-up magnetic resonance imaging (MRI) three months after discharge, showing loss of volume and encephalomalacia in the right and left occipital region representing sequelae of previous infarction

**Figure 5:**
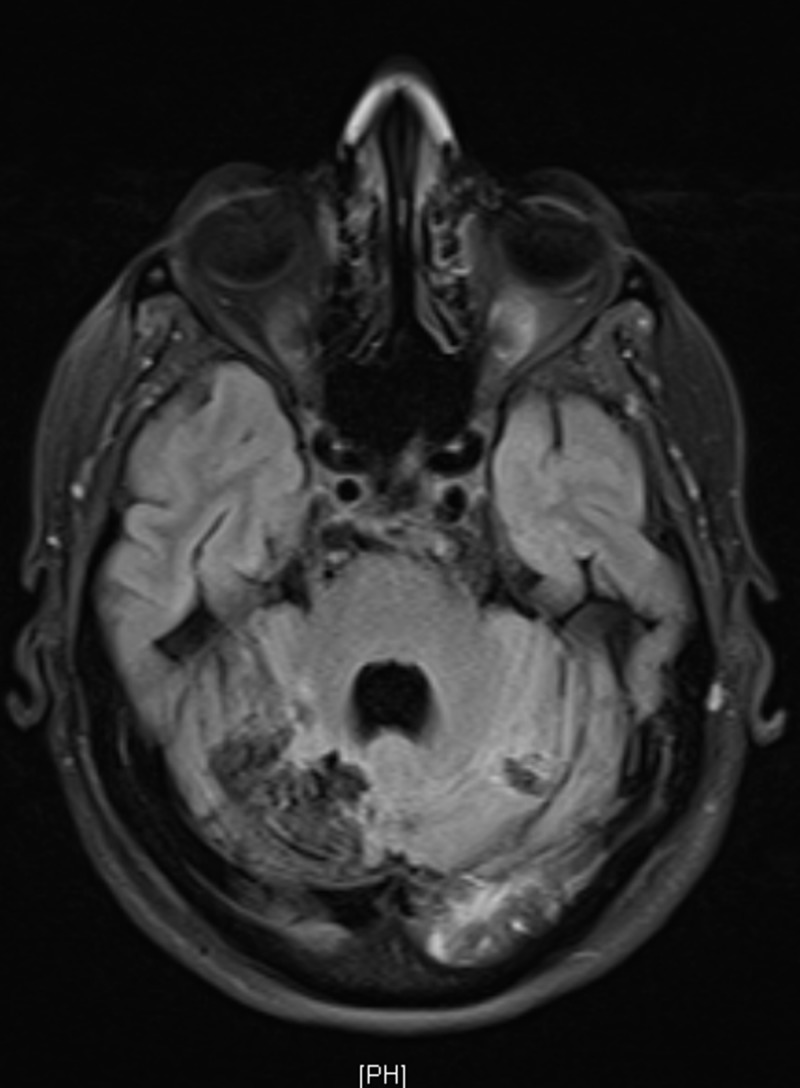
Follow-up magnetic resonance imaging (MRI) three months after discharge showing loss of volume and encephalomalacia in the right and left occipital regions representing sequelae of previous infarction

Upon follow-up in the outpatient clinic after three months, a repeat MRI of the brain was done (Figures 4-5), which revealed a loss of volume and encephalomalacia within the left occipital lobe, cerebellum, and thalamus as well as the corpus callosum with the exo-vacuo lateral ventricle representing sequelae of the previous infarction. It also showed foci of high signal intensity in T2 FLAIR within the white matter and resolution of the left thalamic lesion. Clinically, the patient improved significantly, was completely independent in her activities of daily living (ADLs), and her vision improved significantly to the point that she was planning to return to her job as a teacher.

## Discussion

PRES is recognized as the clinico-radiological manifestations of some complex conditions (pre-eclampsia/eclampsia, allogeneic bone marrow transplantation, organ transplantation, autoimmune disease, high-dose chemotherapy, and immunosuppressive drugs (tacrolimus, cyclosporine, and chemotherapy), Illicit drugs (cocaine), sepsis, and multi-organ failure. The pharmacotoxic side effects of antibiotics can cause PRES as well [3,5]. Two case reports have been published in which antibiotic treatments (ciprofloxacin and linezolid) were identified as the pharmacotoxic cause of PRES [3,5-6]. Some other case studies raise a high suspicion of PRES as a complication after Augmentin and clindamycin ingestion [5].

The underlying pathophysiology of PRES remains elusive. Several theories have been proposed, the most widely accepted of which states that rapidly developing hypertension leads to a breakdown in cerebral autoregulation, particularly in the posterior circulation. Hyperperfusion ensues with protein and fluid extravasation, producing focal vasogenic edema. On the contrary, the hypertension theory is refuted by the fact that PRES can develop in 20%-40% of patients with normal blood pressure or their blood pressure never reaches levels that can disrupt autoregulatory pathways (>150-160 mmHg of the mean arterial pressure) to cause brain swelling [4]. An alternative mechanism implicates endothelial dysfunction as in pre-eclampsia, eclampsia, and sepsis [7]. A third mechanism, which is also significantly discussed in the literature, proposes that vasoconstriction with hypoperfusion subsequently causes ischemia [4].

At CT/MR imaging, the brain typically demonstrates focal regions of asymmetric hemispheric edema. The parietal and occipital lobes are most commonly affected, followed by the frontal lobes, the inferior temporal-occipital junction, and the cerebellum [8]. Lesion confluence may develop as the extent of edema increases. MRI diffusion-weighted imaging (DWI) is instrumental in establishing and consistently demonstrating that the areas of abnormality representing vasogenic edema [8-10].

The clinico-radiologic features are mostly the same whether PRES is due to solid organ tumor, eclampsia/pre-eclampsia, cancer chemotherapy, or significant systemic illness. The process at the molecular level of the disease is also alike, causing T cell activation, endothelial cell activation, and release of endothelin, which contributes to endothelial injury, impaired vascular permeability, and hypoperfusion, in addition to releasing procoagulants like tumor necrosis factor (TNF) alpha and cytokines like IL-1 and gamma interferons. The enhanced release of these factors contributes to vasoconstriction and cerebral hypo-perfusion [11-12]. The radiologic picture of PRES in a subset of patients is more challenging than others. In a study by Fugate et al., the lesions were asymmetric in almost 50% of patients with PRES, 26% of patients showed restriction diffusion while 10% of patients had hemorrhages [3].

The definitive diagnosis of PRES can be made through clinical and radiologic features and the treating physicians should keep these diagnostic features in mind with particular attention towards eliciting a proper drug history and a related medical history from the patient.

## Conclusions

Although the exact etiology of posterior reversible encephalopathy syndrome remains controversial, the diagnosis of PRES should be considered in patients with a compatible clinical history and radiological presentation. Diffusion restriction and evidence of hemorrhage on imaging are usually associated with a poor outcome. Although clinical and radiological features are not reversible in all cases, timely diagnosis and management are of paramount importance to improve the outcome.
